# Function and Mechanisms of Truncated BDNF Receptor TrkB.T1 in Neuropathic Pain

**DOI:** 10.3390/cells9051194

**Published:** 2020-05-11

**Authors:** Tuoxin Cao, Jessica J. Matyas, Cynthia L. Renn, Alan I. Faden, Susan G. Dorsey, Junfang Wu

**Affiliations:** 1 Department of Anesthesiology and Center for Shock, Trauma and Anesthesiology Research (STAR), University of Maryland School of Medicine, Baltimore, MD 21201, USA; tcao@som.umaryland.edu (T.C.); jjanematyas@gmail.com (J.J.M.); afaden@som.umaryland.edu (A.I.F.); 2 Department of Pain and Translational Symptom Science, University of Maryland School of Nursing, Baltimore, MD 21201, USA; renn@umaryland.edu (C.L.R.); sdorsey@umaryland.edu (S.G.D.); 3 Center to Advance Chronic Pain Research, University of Maryland, Baltimore, MD 21201, USA

**Keywords:** tyrosine receptor kinase B (TrkB), TrkB.T1, brain-derived neurotrophic factor (BDNF), astrocytes, neuropathic pain, spinal cord injury

## Abstract

Brain-derived neurotrophic factor (BDNF), a major focus for regenerative therapeutics, has been lauded for its pro-survival characteristics and involvement in both development and recovery of function within the central nervous system (CNS). However, studies of tyrosine receptor kinase B (TrkB), a major receptor for BDNF, indicate that certain effects of the TrkB receptor in response to disease or injury may be maladaptive. More specifically, imbalance among TrkB receptor isoforms appears to contribute to aberrant signaling and hyperpathic pain. A truncated isoform of the receptor, TrkB.T1, lacks the intracellular kinase domain of the full length receptor and is up-regulated in multiple CNS injury models. Such up-regulation is associated with hyperpathic pain, and TrkB.T1 inhibition reduces neuropathic pain in various experimental paradigms. Deletion of TrkB.T1 also limits astrocyte changes in vitro, including proliferation, migration, and activation. Mechanistically, TrkB.T1 is believed to act through release of intracellular calcium in astrocytes, as well as through interactions with neurotrophins, leading to cell cycle activation. Together, these studies support a potential role for astrocytic TrkB.T1 in hyperpathic pain and suggest that targeted strategies directed at this receptor may have therapeutic potential.

## 1. Introduction

Neuropathic pain, which can occur from trauma to or dysfunction of the peripheral or central nervous systems (PNS or CNS), affects approximately 8–10% of the adult population [1], with a cost of more than $40 billion annually. This pain is typically severe and persistent and responds poorly to traditional analgesics, often leading to long-term disability and reduced quality of life. Further, conventional therapeutics, such as opioids, have often limited efficacy in neuropathic pain and can also confer great risk for addiction. Despite modest treatment advances, most patients with neuropathic pain fail to obtain adequate pain relief. In part, this reflects inadequate understanding of the mechanisms underlying the development and persistence of such pain.

Spinal cord injury (SCI) causes not only sensorimotor deficits and autonomic changes, but also chronic, severe, and often unrelenting pain (SCI-pain) that occurs in up to 85% of patients [2,3,4,5]. SCI-pain typically begins weeks to months after injury and may present as increased sensitivity to noxious stimulation (hyperalgesia), pain in response to previously innocuous stimuli (allodynia), and/or spontaneous pain [6,7,8,9,10,11]. SCI-Pain may be diffuse, bilateral, and not necessarily restricted to the lesion level. It has neuropathic features and is often resistant to conventional pain therapies. The delayed expression of SCI-pain, the diffuse localization of painful symptoms, and the presence of pain below the denervated spinal segment suggest maladaptive plasticity not only in the spinal cord, but also in supraspinal structures [12]. Identifying mechanisms responsible for post-injury neuropathic pain may provide new targets for more effective therapeutic interventions.

Brain-derived neurotrophic factor (BDNF), a member of the neurotrophin family of growth factors, modulates pain processing within the CNS [13,14,15,16,17]. Noxious stimulation increases BDNF expression in the spinal dorsal horn (SDH) [18,19,20,21,22] and brainstem [23,24], leading to hyperalgesia and the development of tactile allodynia through enhanced BDNF signaling mechanisms. Increased BDNF expression has been implicated in the development of central sensitization and pain across a range of traumatic and drug-induced persistent pain models [25,26,27,28].

Recently, a truncated isoform of tyrosine receptor kinase B (TrkB) was implicated in the pathobiology of neuropathic pain [29]. Differential splicing during the transcription of TrkB results in three readily identified isoforms, including a full length receptor (TrkB.FL) and two truncated isoforms (TrkB.T1 and TrkB.T2) that are identical to TrkB.FL with regard to the extracellular and trans-membrane regions, but lack the intracellular kinase domain [30,31,32,33]. TrkB.FL is present in multiple cell types and transduces the BDNF signal through several classical pathways; thereby, contributing to normal nervous system development, including myelination and cell survival [34,35,36]. Little is known about the function of TrkB.T2. In contrast, TrkB.T1 has been increasingly studied. It is expressed in multiple cell types [37]; however, importantly, it is the sole isoform expressed by astrocytes [37,38,39,40] and is up-regulated in various experimental models of trauma or pain [29,41,42]. Competitive binding of BDNF to TrkB.FL and TrkB.T1 receptors, along with up-regulation of TrkB.T1, appears to play important roles in the progression of various neuropathic disorders [18,43,44,45], as well as in the development of neuropathic pain after CNS injury [29,41,42].

This review explores the current research on the function and mechanisms of TrkB.T1 in neuropathic pain, including SCI-pain. TrkB.FL-and TrkB.T1-mediated differential signaling pathways are reviewed with regard to both neurodevelopment and neuropathology. Potential mechanism studies addressing the role of TrkB.T1 in nociception and neuropathic pain are highlighted, including the possible role of cell cycle regulation. In addition, we explore the potential cellular mechanisms involved, such as the role of elevated TrkB.T1 expression in astrocytes and post-injury reactive astrogliosis, as well as altered transcriptional programming that controls cellular movement and immune function in relation to injury-induced neuropathic pain.

## 2. BDNF and TrkB Receptor Function during Development

**Figure 1:**
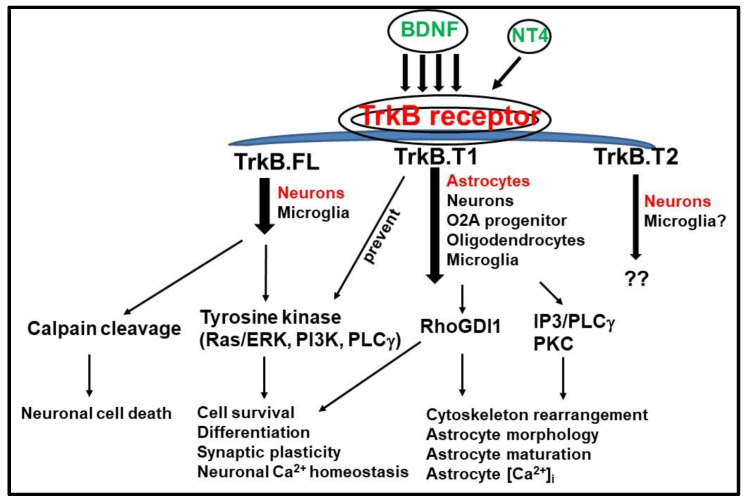
Tyrosine receptor kinase B (TrkB) receptor isoform expression and signaling in the central nervous system. TrkB.T1 is predominantly expressed in astrocytes, whereas TrkB.TL is highly expressed in neurons. Although TrkB.T2 was reported to be expressed by neurons, its function is unknown. Oligodendrocyte-type-2 astrocyte lineage (O2A progenitor). Rho guanosine diphosphate dissociation inhibitor (RhoGDI1).

BDNF and its receptors are important for the development and maintenance of both the PNS and CNS. BDNF plays a critical role in cell growth, differentiation, maturation, morphology, and synaptogenesis through its receptors [46,47,48]. For example, BDNF induces neurogenesis in the subventricular zone (SVZ) of the olfactory bulb; whereas, mice deficient in either BDNF or the TrkB.FL receptor show cell loss in the SVZ hippocampus, cortex, and cerebellum [40,49,50,51] (Figure 1). Activation of TrkB.FL by BDNF is neuroprotective, promoting cell survival and synaptic plasticity in experimental SCI and traumatic brain injury (TBI) [49,52,53,54], while mutations in TrkB.FL are associated with neurological disorders, such as schizophrenia, posttraumatic stress disorder, Parkinson’s disease, Alzheimer disease, and Huntington’s disease [55,56,57,58,59,60].

The most common isoform of TrkB is TrkB.FL, which is the primary receptor for BDNF [29,61]. The intracellular C-terminus of TrkB.FL contains tyrosine-rich catalytic regions and binding sites for phospholipase C gamma (PLCγ) and proto-oncogene tyrosine-protein kinase Src homology 2 domain containing (Shc) [62]. Through high-affinity binding to the extracellular domain of TrkB.FL, BDNF induces autophosphorylation of the receptor to expose the binding domains for PLCγ, adenosine triphosphate (ATP), Shc, suc1-associated neurotrophic factor target (SNT) protein, and growth factor receptor-bound protein 2 (GRB2). Activation of PLCγ catalyzes the phosphatidylinositides to inositol trisphosphate (IP3) and diacylglycerol (DAG) [63]. Subsequently, IP3 causes a release of calcium from endoplasmic reticulum and DAG activates protein kinase C (PKC) to promote neuroplasticity and cell survival [63,64]. Secondly, TrkB autophosphorylation can lead to neurite outgrowth, neuron survival, and dendric outgrowth through binding of Shc and GRB2 and activation of retrovirus-associated DNA sequences (Ras) and the phosphatidylinositol 4,5-bisphosphate 3-kinases/protein kinase B (PI3K/Akt) pathway [47,64]. Finally, binding of Shc also regulates the extracellular signal-regulated kinase/mitogen-activated protein kinase (Erk/MAPK) pathway and promotes differentiation and proliferation [62,63].

Alternative splicing of TrkB produces the TrkB.T1 isoform, which has a truncated C-terminal domain [30,31,32]. As TrkB.T1 lacks the intracellular domains of TrkB.FL, autophosphorylation is lost and it cannot bind to Shc and PLCγ. Instead, TrkB.T1 has the activation juxtamembrane sequence domain (KFG) (the lysine-phenylalanine-glycine amino acid sequence) [61]. A 3 amino acid KFG (mouse residues 450–452, corresponding to human residues 441–443) is conserved among all Trk genes, suggesting an important biological function [65]. Recent studies demonstrated that the TrkA KFG mutant negatively regulates TrkA protein level and function in response to nerve growth factor (NGF) [65]; moreover, mutant mice show enhanced thermal sensitivity and inflammatory pain [66]. There are two binding sites that can activate MAPK related signaling pathways on the TrkB.FL, the traditional Shc binding site (Y490) and the KFG domain, where fibroblast growth factor receptor substrate 2 (FRS2)/SNT can bind. Negative regulation through the TrkA KFG mutant is likely associated with the KFG domain on the full-length isoform of TrkA. Although the KFG domain is conserved and present on the truncated TrkB.T1 isoform, a role for this domain in TrkB.T1-mediated signaling has not been described.

TrkB.T1 is expressed at high levels during key points of neurodevelopment, including the formation of synapses and remodeling of axons [35] and the sprouting of dendritic filipodia [67]. In the PNS, TrkB.T1 expression correlates strongly with myelination [34]. The beneficial effects of TrkB.T1 continue into adulthood through its role in the development of plasticity, formation, and restructuring of the actin cytoskeleton and in astrocyte morphology in the neocortex [36,38]. For example, a recent study [37] demonstrates a critical role for BDNF/TrkB.T1 signaling in astrocyte morphological maturation during CNS development. However, the beneficial effects of TrkB.T1 predominantly occur at normal physiological levels; whereas, increased levels of TrkB.T1 expression are commonly found in response to injury or illness. Such up-regulated expression of TrkB.T1 appears to have adverse effects. In addition, both full-length and truncated TrkB receptors are highly expressed in developing trigeminal ganglion [68], which is the main origin of the cerebrovascular sensory nerve fibers. Whether or not these TrkB isoforms play a critical role in developing a sensory system is unclear.

## 3. TrkB.FL and TrkB.T1 Differential Signaling Pathways

### 3.1. TrkB.T1 Isoform as an Inhibitor of TrkB.FL

With only 11 amino acids in the intracellular domain, TrkB.T1 cannot transduce signals through classical intracellular pathways [29,32]. However, with an extracellular domain that is identical to TrkB.FL, TrkB.T1 binds to BDNF with the same affinity. Both TrkB.FL and TrkB.T1 are found in a number of brain regions [47]. TrkB.T1 is expressed in neurons and more highly expressed in oligodendrocyte-type-2 astrocyte lineage (O2A progenitors), astrocytes, and oligodendrocytes [69,70]. TrkB.T1 also forms heterodimers with TrkB.FL. The TrkB.FL-TrkB.T1 heterodimer, the TrkB.FL-TrkB.FL homodimer, and the TrkB.T1-TrkB.T1 homodimer all occur in the primate brain [71]. As TrkB.T1 lacks the intracellular catalytic domain, TrkB.FL-TrkB.T1 heterodimers cannot undergo cross-autophosphorylation between the two receptors. Thus, TrkB.T1 can function as a dominant negative inhibitor of the TrkB.FL and prevent subsequent activation of PI3K/Akt, Erk/MAPK, and PLCγ pathways [72,73]. Therefore, increased expression of TrkB.T1 can prevent BDNF mediated cell proliferation and cell survival [73].

TrkB.T1 can also bind and internalize BDNF without activation of classical TrkB.FL pathways, which allows TrkB.T1 to bind, sequester, and then store BDNF in transport vesicles. In early chick development, it was found that picomolar concentrations of BDNF can be rapidly internalized by TrkB.T1 expressing cells and limit activity and diffusion of BDNF [74]. Indeed, transfection and expression of TrkB.T1 in 3T3 fibroblast cells prevented neurite outgrowth of a co-cultured SY5Y neuronal cell [75]. This inhibition by TrkB.T1 expression can be reversed by the addition of high concentration of BDNF, indicating that inhibition is not due to blocking the TrkB.FL receptor on SY5Y cells directly [75]. CNS astrocytes, which only express TrkB.T1, can sequester and subsequently re-release BDNF from transporting vesicles [76]. This suggests that TrkB.T1 may also function as a temporal buffer to reduce BDNF concentration when it is too high.

### 3.2. TrkB.T1 Signaling Pathway Independent of TrkB.FL

Traditionally, TrkB.T1 has been thought to be a dominant negative inhibitor of TrkB.FL by competing with its ability to bind to BDNF. However, recent studies in neurons and astrocytes suggest that TrkB.T1 acts through the modulation of signaling pathways different from those of TrkB.FL. Although TrkB.T1 lacks the catalytic tyrosine kinase domain and the Shc, ATP, or PLCγ binding domains, TrkB.T1 does have a potential SNT protein binding site at the KFG sequence [77].

One of its identified binding proteins is the rho GDP dissociation inhibitor (RhoGDI1) [35]. RhoGDI1 inhibits Rho hydrolase enzymes of the nucleotide guanosine triphosphate (GTPase), which dissociates from TrkB.T1 upon binding to BDNF and modulates glycine transporters in astrocytes [35,78]. This intracellular release of RhoGDI1 can cause alterations in cell morphology by serving as a negative inhibitor of the Rho signaling cascade, which in turn remodels the actin cytoskeleton through p21-activated kinase (PAK) and MAPK signaling cascades [36]. Furthermore, TrkB.T1 mediates calcium influx in astrocytes [39], whereas TrkB.FL contributes to neuronal Ca^2+^ signaling [79,80]. The BDNF-induced [Ca^2+^]i in astroglial cells do not require TrkB.FL [39]. The function of TrkB.T1 in neurons, which express both full-length and truncated TrkB isoforms, is more complex. Although complete depletion of TrkB.T1 does not affect resting [Ca^2+^]i in neurons, proper TrkB.T1 levels (or the relative ratio of TrkB.FL to TrkB.T1) are critical for maintaining neuronal Ca^2+^ homeostasis [40]. TrkB.T1 in astrocytes mediates IP3–dependent calcium release from intracellular stores. Current speculation on underlying mechanisms is that activation of TrkB.T1 by BDNF activates yet unidentified G-proteins, which induce PLCγ activation of IP3 and intracellular calcium release in astrocytes similar to that of TrkB.FL [39,81]. This is unusual as TrkB.T1 has short intracellular carboxy-terminal tails and no intrinsic catalytic activity [30,82]. It is likely that additional interacting factors are required, such as other membrane-spanning receptors or truncated TrkB interacting protein, which has been identified to bind TrkB.T1 [83]. TrkB receptors can be activated by ligands of the G-protein-coupled receptor (GPCR) family of transmembrane receptors in the absence of BDNF [84,85]. Whether or not TrkB.T1-induced PLCγ activation of IP3 through activation of GPCR remains unknown. This change in intracellular calcium concentration also may contribute to the rearrangement of the actin cytoskeleton and filopodia outgrowth [35,36,39]. Thus, overexpression of TrkB.T1 decreases cell proliferation but increases numbers of filopodia and distal dendritic elongation [86]. However, TrkB.T1 does not appear to require BDNF binding to induce distal dendritic elongation; rather, it may inhibit BDNF binding-induced TrkB.FL-mediated proximal dendritic growth [86]. TrkB.T1 also activates PKC signaling through increased phosphorylation of the myristoylated alanine-rich C-kinase substrate (MARCKS), the most prominent cellular substrate for multiple isoforms of PKC, and therefore, promotes brain cells to assume astrocyte-like morphology [87]. Finally, TrkB.T1 knockout (KO) mice show reduced cell cycle proteins, suggesting that TrkB.T1 contributes to activation of cell cycle pathways [41]. We have previously reported [40,41] that TrkB.T1 deletion in the KO mice does not affect full-length TrkB expression evidenced by western blot. However, whether or not depletion of TrkB.T1 disturbs TrkB other isoforms in different cell types is unclear.

Therefore, active signaling by TrkB.T1 has profound and varying effects. Targeted deletion of TrkB.T1 in muscle that has been subjected to acute injury, showing increases in neurotrophin-dependent activation of Akt, a downstream signaling target of TrkB.FL, and subsequently improved neuromuscular function [32]. In the trisomy 16 mice, the reduction of elevated TrkB.T1 levels restores BDNF-induced neuronal survival [40]. TrkB.T1 null mice subjected to experimental SCI show improved recovery as compared to wild-type mice, with up-regulated levels of TrkB.T1 expression and reduced expression of cell cycle proteins, such as cyclin-dependent kinase 4 (CDK4) and proliferating cell nuclear antigen (PCNA) [41]. Overall, these findings support the concept that TrkB.T1 functions through non-traditional intracellular signaling mechanisms, which include triggering the release of calcium in astrocytes, activating the cell cycle and inhibiting proteins that affect the structure of cells. Therefore, TrkB.T1 may be a potential therapeutic target for a number of neurological disorders in which the latter signaling pathways have been implicated.

## 4. Function and Mechanisms of TrkB.T1 Receptor in CNS Insults

### 4.1. TrkB.T1 in Neurological Disorders

Although a number of neurotrophins, including BDNF, are up-regulated in response to CNS insults, the up-regulation of truncated TrkB receptors participates in the inhibition of axonal regeneration, likely through competitive binding in the extracellular space and at the cell membrane [31,75]. The TrkB.T1 isoform is highly conserved across species, both in its genetic sequence and its high-affinity binding with BDNF, and does not appear to affect the expression patterns of TrkB.FL [40,81]. TrkB.T1 is expressed in both neurons and glia. However, neurons primarily express the TrkB.T2 truncated isoform, whereas astrocytes express TrkB.T1 exclusively [35,38,39,78,88]. TrkB.T1 upregulation is common in multiple neurological disorders, and such upregulation activates cell cycle pathways, which can cause neuronal cell death, astrogliosis, and microglial proliferation/activation [89] and inhibit cell regeneration and repair [41]. In a rodent model of trisomy 16, neurons containing a duplicate chromosome 16 were found to overexpress TrkB.T1 compared to unaffected cells and contributed to premature cell death in hippocampal neurons [40]. Neuronal survival in those cells was found to improve when TrkB.FL expression was increased, as well as in response to genetic deletion of TrkB.T1.

In other neurodegenerative disease models, increased TrkB.T1 expression also correlates with disease progression. For example, expression levels of BDNF and TrkB.FL are decreased, whereas TrkB.T1 is increased in Alzheimer’s disease [44], with suggestions that this change may at least partially be mediated by amyloid-beta plaques in a calpain-dependent mechanism [90]. Alzheimer’s disease models also show a correlation between TrkB.T1 elevation and disease progression, although whether this correlation reflects a reduction of TrkB.FL signaling or increased TrkB.T1 signaling is not fully understood [44]. However, other studies indicate that amyloid beta exposure can increase TrkB.FL expression in cultured SHSY5Y neuroblastoma cells but reduced TrkB.T1 expression [91]. TrkB.FL increases the amyloid precursor protein (APP) intracellular domain (AICD)-mediated gene transcription and APP levels in SHSY5Y cells decreasing soluble APP levels, but TrkB.T1 did not affect APP metabolism [92]. When TrkB.T1 is co-transfected with TrkB.FL, the latter effect on APP metabolism is lost, suggesting that these actions are mainly mediated by the tyrosine kinase activity of the TrkB.FL [92]. In human Parkinson’s disease patients, the distribution of TrkB.FL and TrkB.T1 are even more complex. TrkB.FL is increased in striatal neurons and substantia nigra pars compacta (SNpc) axons but decreased in striatal neurites, SNpc somata, and dendrites [70]. On the other hand, TrkB.T1 is increased in striatal soma and SNpc distal dendrites but decreased in striatal axons [70]. Although the changes in TrkB isoform expression in SN regions and its effect on Parkinson’s disease are therefore not fully understood, TrkB.FL and/or TrkB.T1 may be a potential drug target, as BDNF is neuroprotective and may be beneficial for Parkinson’s disease [93,94].

In amyotrophic lateral sclerosis (ALS), BDNF has long been a drug candidate. Both in vivo and in vitro models show that BDNF, as well as BDNF analogs and TrkB agonists, such as 7,8-Dihydroxyflavone, can prevent motor neuron cell death and development of ALS [95,96]. However, phase III clinical trials failed to demonstrate significant benefits of BDNF treatment in ALS patients. TrkB mRNA and protein levels are increased, whereas the phosphorylated TrkB receptors are reduced [97]. One possibility is that increased TrkB.T1 prevents TrkB.FL cross autophosphorylation. Indeed, TrkB.T1 KO mice showed enhanced hippocampal Cornu Ammonis (CA1) parvalbumin-positive neuron survival and delayed onset of disease symptoms such as motorneuron degeneration and development of muscle weakness in a mouse model of ALS [98,99]. Furthermore, application of adenosine A2A receptor agonist CGS21680, a drug that phosphorylates TrkB.FL in motoneurons, regardless of the presence of BDNF or TrkB.T1, slowed the onset of ALS, similar to TrkB.T1 deletion in mice, supporting the concept that increased TrkB.T1 prevents BDNF stimulation through inhibition of TrkB.FL activity [99]. These results support the concept of an imbalance between TrkB isoform expression patterns as a potential factor in ALS pathobiology.

### 4.2. TrkB.T1 in Traumatic Spinal Cord Injury and Brain Injury

Following moderate contusion injury in mice, the upregulation of TrkB.T1 expression is observed at 24 h post-injury, with sustained elevation in white matter through 8 weeks [42,100]. TrkB.T1 protein levels are significantly increased in astrocytes and microglia in white matter and neurons in gray matter [41,42,101]. In a spinal cord hemi-section model TrkA, TrkB.FL, and TrkC receptors were not present in the lesion site but were expressed in uninjured parts of the spinal cord; whereas, the TrkB.T1 receptor was expressed at the injury border and within the glial scar. P75 receptors are expressed on Schwann cells throughout the lesion site [69,102]. BDNF-facilitated the α-amino-3-hydroxy-5-methyl-4-isoxazolepropionic acid (AMPA) receptor, and N-methyl-d-aspartate (NMDA) current is reduced after SCI [103]. Furthermore, the cell cycle proteins, CDK4, PCNA, and transcription E2 factor 5 (E2F5), are significantly increased in tissues that showed concomitant TrkB.T1 up-regulation [41]. When treated with CR8, an inhibitor of CDKs, these proteins were reduced to levels found in TrkB.T1 null mice [41]. Deletion of TrkB.T1, as well as treatment with CR8, improved functional recovery from SCI in mice, increased spared white matter in the lesion region, and reduced gliosis, which is associated with neuropathic pain below the injury level [41]. These findings suggest that TrkB.T1 has a mechanism of action upstream of cell cycle regulation. In addition, RNA sequencing of astrocytes to examine differential gene expression in cells with or without TrkB.T1 demonstrate significant decreases in pathways related to inflammation, cellular proliferation, and migration in the absence of TrkB.T1. This finding has been validated in functional assays, both in vitro and in vivo, where deletion of TrkB.T1 in astrocytes reduces cell proliferation and migration [41,42]. Taken together, these findings suggest that TrkB.T1 depletion may affect astrocyte responsiveness to injury and reduce the formation of the astroglia scar.

Similar to SCI, TrkB.T1 receptor expression is increased in the brain after injury. In the rat hippocampus, following ibotenic acid-induced neuronal injury, TrkB.T1 mRNA is elevated with a time-course similar to glial fibrillary acidic protein mRNA expression increases [104]. Traumatic injury to the striatum or excitotoxic kainic acid injury also increase the expression of TrkB.T1 for as long as 2 months without changing TrkB.FL receptor expression [105,106,107]. Similar changes are found in a brain ischemia model, where TrkB.T1 is increased in astrocytes surrounding the infarction, whereas TrkB.FL is reduced in the infarct region [108]. These results suggest that TrkB.FL-related functions, such as neuronal survival and proliferation, are suppressed, whereas TrkB.T1-related effects, such as sprouting and cell mobility, are increased following brain injury. BDNF administration did not affect regeneration or cell survival, but promoted axonal sprouting of serotonergic neurons after neurotoxic injury [109]. The imbalance of TrkB.FL to TrkB.T1 levels is also thought to underlie cell death related to neurotoxicity. Consistent with this perspective, overexpressing TrkB.T1 exacerbates neuronal loss in a model of transient focal cerebral ischemia [110], whereas increasing TrkB.FL levels or reducing TrkB.T1 expression protect neurons from excitotoxic death [111]. In addition, TrkB.T1 protein levels are reduced after ischemic pre-conditioning [112]. These results support the view that TrkB.T1 activation can exacerbate neuronal injury through inhibition of TrkB.FL. However, the effect of TrkB.T1 in CNS injury may not be simple, as activation of TrkB.T1 receptors can also lead to inhibition of RhoA, which in turn reduces excitotoxic signaling [113]. Further research is therefore needed to elucidate the effects of TrkB.T1 signaling in CNS injury and recovery independent of TrkB.FL.

### 4.3. BDNF/TrkB.T1 Regulation in Neuropathic Pain

Pain pathways in the adult spinal cord are primarily processed within the superficial SDH. After SCI and peripheral nerve injury, neurons in the SDH exhibit increased spontaneous activity, reduced activation thresholds, and an increased response to suprathreshold stimulation [11,114]. Neuropathic pain that occurs following various types of nerve injury may result, at least in part, from loss of inhibitory interneurons, leading to hyperactive signaling in cells that would otherwise only respond to painful stimuli [115]. However, less clear is the role for BDNF/TrkB.T1 signaling in the regulation of SDH neuronal hyperactivity following neuropathic pain.

**Figure 2:**
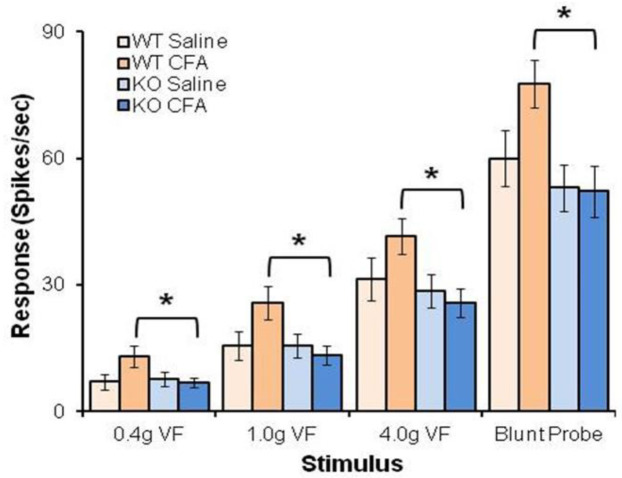
TrkB.T1 deletion decreases wide dynamic range neuron hyperexcitability after inflammation. Complete Freund’s Adjuvant (CFA, 20 µL) or saline was injected subcutaneously into the plantar surface of the left hind paw in both TrkB.T1 WT and knockout (KO) mice. After 24 h, wide dynamic range neurons (WDRNs, the number of spikes per second) were recorded in the lumbar enlargement spinal dorsal horn (SDH) ipsilateral to the inflammation. In response to stimulation of von frey filament (VF) and blunt probe, there was significantly less neuronal hyperexcitability of neurons from inflamed KO mice compared to WT. There was no difference between saline-treated WT and KO mice (*n* = 15 per group). * *p* < 0.05 WT CFA vs KO CFA with two-way analysis of variance (ANOVA), following Tukey’s multiple comparisons test.

Antiretroviral treatment causes mechanical allodynia in the mouse by increasing BDNF expression, which leads to hyperexcitability of wide dynamic range (WDR) neurons in the dorsal horn [26]. However, few studies have examined the role for TrkB.T1 in nociception. Yajima et al. [28] administered intrathecal anti-TrkB.T1 (Santa Cruz Biotechnology, Santa Cruz, CA, USA) and failed to observe any change in thermal hyperalgesia in nerve-injured mice. However, this antibody has been shown to bind to epitopes other than TrkB.T1, making the interpretation of this study difficult. We examined TrkB isoform mRNA and protein expression in the SDH of mice treated with an antiretroviral drug or a hind paw injection with Complete Freund’s Adjuvant (CFA) and found that TrkB.T1 was up-regulated, whereas TrkB.FL levels were unchanged [29]. Similar findings were also reported in the dorsal root ganglia of antiretroviral-treated mice [116]. To increase our understanding of the function of TrkB.T1, we developed a TrkB.T1-specific knockout mouse [40]; the absence of TrkB.T1 reduced the development of allodynia and thermal hyperalgesia in these model systems, respectively [29]. As we [26] and others [117] have demonstrated that the BDNF increase in SDH following chemotherapy or surgical nerve injury produces hyperexcitability of WDR neurons in the dorsal horn, we therefore examined whether there were differences in neuronal excitability after hind paw inflammation in TrkB.T1 null versus wildtype (WT) mice. As shown in Figure 2, WDR neuronal excitability in the CFA-treated KO mice was no different than vehicle-treated WT mice. Only drug-treated WT mice displayed increased neuronal excitability. These results suggest that the analgesic effects of TrkB.T1 deletion (e.g., decreased allodynia and thermal hyperalgesia) are mediated through reduced or absent SDH neuronal excitability. Taken together, these findings suggest that BDNF/TrkB.T1 signaling is responsive to persistent noxious stimulation and promote hyperalgesia and allodynia through the development of hyperexcitability of WDR neurons in the SDH.

With respect to SCI-pain, there is evidence from rodent studies that central sensitization mechanistically contributes to sensory abnormalities and persistence of central neuropathic pain [11,118,119,120]. Among the types of pain that occur after SCI is allodynia and hyperalgesia [121,122]. In animal models of SCI, behaviors reflecting these types of pain can be detected using the von Frey filament test as early as 2 weeks post-injury and continuing well into the chronic phase of recovery. Elimination of TrkB.T1 improved pain-related behaviors in multiple studies of post-SCI pain, including mechanical allodynia, thermal hyperalgesia, and spontaneous pain; it also served to modulate astrocyte morphology and growth [41,42]. However, whether or not depletion of TrkB.T1 reduces SDH neuronal excitability induced by SCI remains unknown. Given the strong evidence for the contributions of TrkB.T1 in the development and maintenance of neuropathic pain, it is important to further address the mechanism of action for TrkB.T1 and its downstream effects.

### 4.4. Astrocytic TrkB.T1 Contributes to Nociception via Dysregulated Cell Cycle Activation

**Figure 3:**
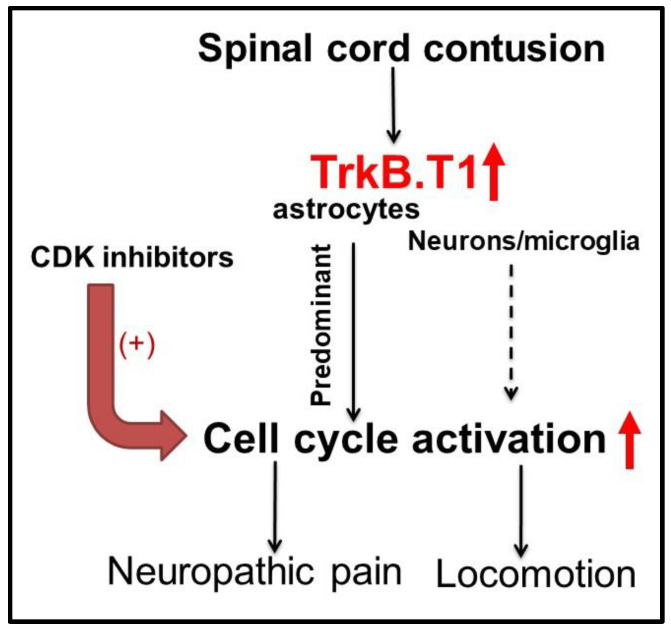
TrkB.T1 contributes to neuropathic pain following spinal cord injury through regulation of cell cycle pathways. Cyclin-dependent kinase (CDK).

To examine the mechanisms underlying the decrease in nocifensive behavior in mice with SCI in the absence of TrkB.T1, a microarray analysis was done to compare gene expression in the spinal cord of TrkB.T1 WT versus KO mice at 24 h, 3 days, and 7 days after moderate contusion injury SCI [41]. The majority of differentially expressed genes at 24 h after injury were related to the cell cycle and DNA damage in only the WT mice. These results are consistent with our previously published studies, in which we reported that cell cycle genes were up-regulated in the spinal cord following SCI, contributing to secondary damage and cell death [123,124]. Pathway analysis of those significantly regulated genes at 24 h after injury show that cell cycle and DNA damage canonical signaling pathways are enriched in the dataset. Comparing the KO to the WT, most of these pathways are down-regulated in the TrkB.T1 KO spinal cord as compared to the WT mice. Whole genome analysis, confirmed at the protein level, revealed that cell cycle genes were up-regulated in WT but not the TrkB.T1 KO spinal cord after SCI [41]. In vitro transforming growth factor (TGF) β-induced reactive astrocytes from WT mice showed increased cell cycle protein expression that was significantly reduced in astrocytes from TrkB.T1 KO mice that express neither full-length TrkB nor TrkB.T1. Administration of CR8, which selectively inhibits CDKs, reduced hyperesthesia, locomotor deficits, and lumbar SDH glial changes after SCI, similar to TrkB.T1 deletion. In TrkB.T1 KO mice, CR8 had no effect. These data indicate that TrkB.T1 contributes to the pathobiology of SCI and SCI-pain through modulation of cell cycle pathways (Figure 3).

In peripheral nerve injury or inflammatory pain models, inhibition of cell cycle activation by the CDKs inhibitors, flavopiridol and roscovitine, reduce hyperalgesia to heat and tactile allodynia [125,126,127]. To examine more directly whether changes in cell cycle signaling pathways are involved in post-SCI-pain, we compared the effects of early vs. late treatment with flavopiridol on allodynia and motor function [128]. Early flavopiridol treatment significantly reduced sensitivity to mechanical and thermal stimulation and locomotor dysfunction. SCI causes robust and extended spontaneous pain using the mouse grimace scale (MGS) [128]. Early administration of flavopiridol significantly shortens duration of MGS changes. Late flavopiridol intervention significantly limited hyperesthesia at 7 days after treatment, associated with reduced glial changes, but without effect on locomotion. Similar findings with the more selective CDK inhibitor CR8 were also found in a rat spinal cord contusion model [129]. Moreover, the ablation of key cell cycle activation components E2F1-2 limits hyperesthesia after SCI [130].

Activated microglia after SCI has been implicated in the maintenance of chronic hyperalgesia [131,132], whereas chronic pain after SCI and peripheral nerve injury may also be mediated through astrocytes [133,134]. Astrocytes form physically coupled networks mediated by gap junctions, which facilitate intercellular transmission of Ca^2+^ signaling, with oscillations in ion permeability through astrocytic networks [135]. Following SCI, astrocytes undergo morphological changes and show enhanced proliferation and migration and increased synthesis of glial fibrillary acidic protein (GFAP) [136,137]. The glial scar can serve as a protective role but can also limit axon regeneration [136,137,138,139]. Reactive astrocytes in the spinal cord have been implicated in the development and maintenance of central sensitization and pain hypersensitivity. Receptor-mediated increases in astrocytic Ca^2+^ can modulate neural network activity and activate other types of glia cells through the production of cytokines, chemokines, and growth factors [11,133,134]. Compared to microglial activation, astroglial activation in neuropathic pain conditions is more persistent and may play a more important role in neuropathic pain maintenance [135,140].

In a mouse spinal cord contusion model [41,42], elevated TrkB.T1 expression was predominantly found in astrocytes. GFAP^+^ astrocytes were reduced in the absence of TrkB.T1 at both the lesion area and the lumbar SDH. RNA sequencing of cultured astrocytes derived from TrkB.T1 KO and WT mice revealed down-regulation of migration and proliferation pathways in KO astrocytes. KO astrocytes also exhibited slower migration/proliferation in vitro in response to 10% serum or BDNF as compared to WT astrocytes. Reduced proliferation of astrocytes was confirmed after SCI in astrocyte specific TrkB.T1 KO mice, which also showed reduced hyperpathic responses in mechanical allodynia and pain-related measurements on the CatWalk, along with improved motor coordination [42]. The association with decreased hyperpathia after injury suggests that TrkB.T1-mediated astrocyte dysfunction contributes to SCI-pain.

## 5. Conclusions and Perspectives

Despite being identified as a contributor to apoptosis and pain signaling, ablation of truncated BDNF receptors is problematic, as the complete elimination of TrkB can have severe detrimental effects. BDNF and its primary receptor TrkB are crucial to nervous system development and serve a protective function in adulthood, contributing to neuronal survival, maintenance of the central and peripheral nervous systems, and facilitating recovery from trauma. However, its truncated receptor TrkB.T1 has harmful effects when up-regulated in response to injury and inflammation, including but not limited to activation of cell cycle proteins, inhibiting axonal repair and facilitating the development of neuropathic pain. Experimental deletion of the TrkB.T1 receptor has successfully reduced the detrimental effects while improving functional recovery from injury and ameliorating pain. Some clues regarding the mechanism of action for TrkB.T1 signaling have been detected and, although more work is needed to fully understand the pathways involved, the truncated receptor appears to be a potential therapeutic target for a number of neurological disorders and for neuropathic pain. In the future, a greater understanding of the mechanistic pathways of TrkB.T1 will hopefully serve to narrow the focus of such therapeutic targeting so that the deleterious effects of the TrkB.T1 pathway are minimized and the favorable effects of BDNF are conserved or amplified.
